# How to Increase the Efficacy of Immunotherapy in NSCLC and HNSCC: Role of Radiation Therapy, Chemotherapy, and Other Strategies

**DOI:** 10.3389/fimmu.2018.02941

**Published:** 2018-12-12

**Authors:** Valerio Nardone, Pierpaolo Pastina, Rocco Giannicola, Rita Agostino, Stefania Croci, Paolo Tini, Luigi Pirtoli, Antonio Giordano, Pierosandro Tagliaferri, Pierpaolo Correale

**Affiliations:** ^1^Radiation Oncology Unit, University Hospital of Siena, Siena, Italy; ^2^Medical Oncology Unit, Grand Metropolitan Hospital “Bianchi Melacrino Morelli”, Reggio Calabria, Italy; ^3^Sbarro Health Research Organization, Temple University, Philadelphia, PA, United States; ^4^Department of Biology, College of Science and Technology, Temple University, Philadelphia, PA, United States; ^5^Department of Medicine, Surgery and Neurosciences University of Siena, Siena, Italy; ^6^Department of Experimental and Clinical Medicine, Magna Graecia University, Catanzaro, Italy; ^7^Medical Oncology Unit, Azienda Ospedaliero – Universitaria “Mater Domini”, Catanzaro, Italy

**Keywords:** NSCLC, radiation therapy, immunotherapy, HNSCC, chemotherapy

An extraordinary large amount of strategies potentially able to elicit and empower an efficient anti-tumor immune-response in cancer patients, has already been described (1). However, a number of hurdles have delayed the translation of these results in efficacious treatments for many years leaving the immunological treatments confined to malignant melanoma and renal cell carcinoma(2, 3). In the latter few years, the discovery of priming (CTLA-4/B7.1) and effector (PD-1/PDL-1) immune-checkpoints and the availability of highly specific blocking mAbs has lead to a terrific clinical development of the immune-oncology approaches. Some of these mAbs, especially those directed to PD-1 (Nivolumab and Pembrolizumab) expressed on activated CTLs, or PDL-1 (Atezolizumab, Durvalumab, and Avelumab) expressed on inflammatory and cancer cells, have in fact, gained a stable role in the treatment of very common malignancies such as non-small cell lung cancer (NSCLC), head and neck squamous cell carcinoma (HNSCC), and urological malignancies, where they are capable of producing significant benefit to many patients and prolonging their survival in about a quarter of the cases (4). Even though this kind of strategy is considered quite successful, it is however, hampered by the fact that its efficacy is unpredictable and is associated to immune-related adverse events (irAEs) and unsustainable costs. At the present, the identification of reliable biomarkers of response to immune-oncology treatments as well as the design of combined strategies to enhance their efficacy and field of action represent one of the mainstream immune-oncology research lines. PD-1/PDL-1 is a peripheral immune-checkpointaimed to attenuate the cytotoxic response of tumor-specific infiltrating lymphocytes. Thus, its blockade by anti PD-1 (Nivolumab and Pembrolizumab) or anti PDL-1 mAbs (Atezolizumab, Durvalumab, and Avelumab) rescues these CTLs and triggers a fast cytolytic effect in the tumor tissue (5). This effect may triggera rapid antitumor effect;neverthelessthis renewed CTL reaction is not sufficient alone to prolong patients' survival. In fact the antitumor activity of these reactivated cells, is more or less rapidly extinguished if a continuous and self-sustained supply of fresh tumor-specific immune-effectors does not occur (immunopriming) (6). Experimental evidence suggests in fact, the achievement of a prolonged patient survival requires a continuous immune-priming, in order to avoid CTL exhaustion in the tumor and to prevent an adaptive response by the tumor cells (7, 8). In this context, CTLA-4/B7.1 immune-check point, acts by attenuating the proliferative activity of antigen specific CTL clones, expressing CTLA-4 and by stimulating the immune-suppressive activity of immune-regulatory T cells (T_reg_s). Its blockade by Ipilimumab and Tremelimumab, two mAbs to CTLA-4, represents a valid therapeutic option for both metastatic malignant melanoma and renal cell carcinoma and is under clinical investigation in combination with effector PD1/PDL-1 immunocheckpoint blockade (9–12). An efficient Immune-priming however, requires the expression of multiple tumor associate (TAAs) and tumor specific antigens (TSAs) by cancer cells, released as consequence of cancer-associated inflammation, necrosis, previous use of cytotoxic drugs or radiation therapy (13). A number of studies have shown that the efficacy of both immune-effectors and antigen cross-priming may be hardened by cancer vaccines, specific anticancer treatments (radiotherapy, chemotherapy, steroid hormones, and immune-adjuvant agents), hypoxic response and/or tumor associated inflammation (14, 15) (Figure 1).

**Figure 1:**
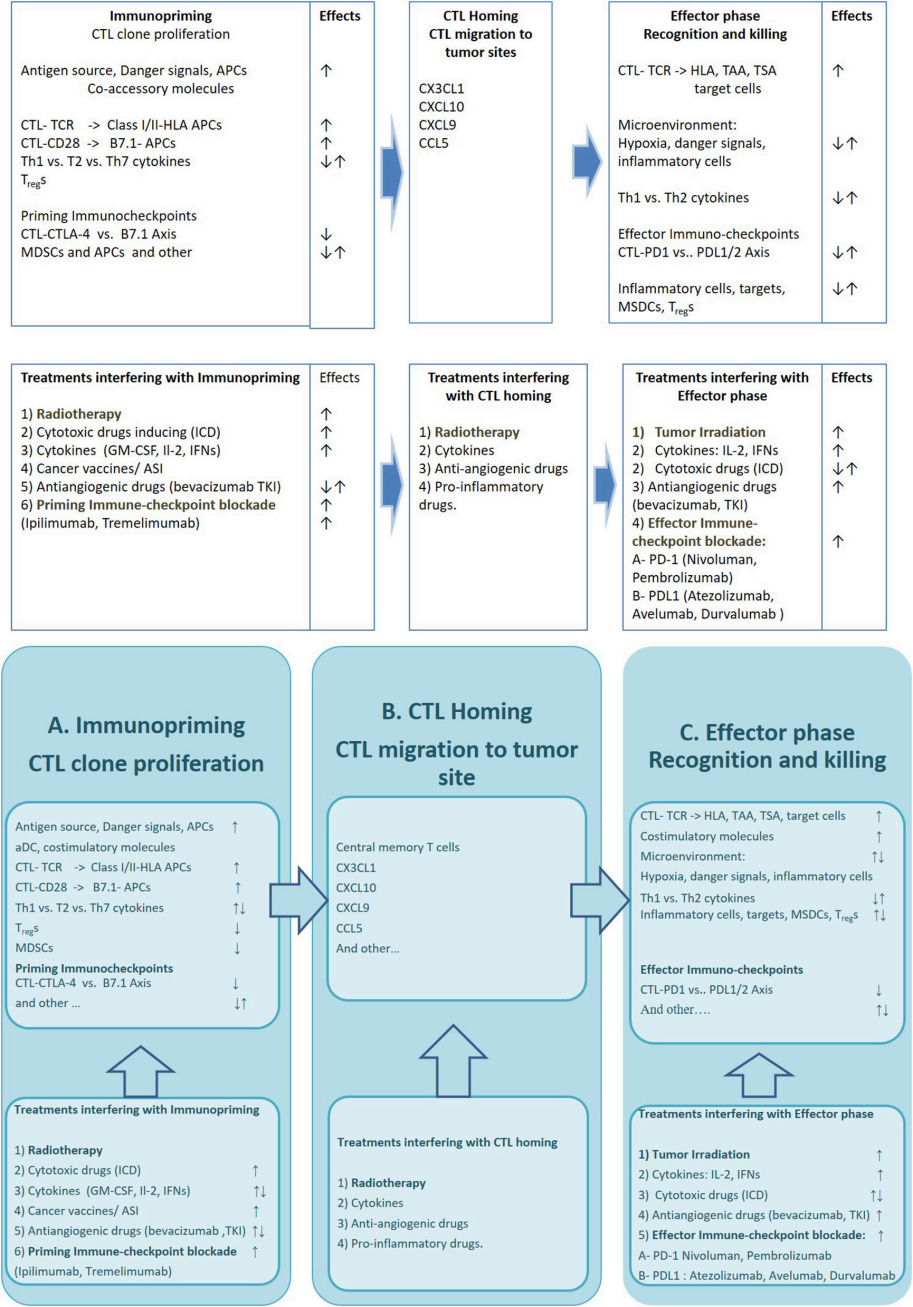
The figure describes the critical mechanisms involved in three phases of the immune-response against cancer and available drugs and strategies which may improve its efficacy **Upper row:** Specific cell lineages, molecular structures and immune-checkpoints involved in immunopriming process (A), T cell Homing (B), and modulation of CTL mediated Tumor cell killing (C). **Bottom row:** Strategies (AKA radiation therapy), cytotoxic Drugs, cytokines and Immunocheckpoint inhibitors interfering with the immunopriming (A), T cell Homing (B), and T cell mediated killing (C). APCs, antigen presenting cells; CTL-TCR, cytotoxic T lymphocites–T cell Receptor; HLA, Human Leucocyte Antigen; MDSCs, myeloid derived suppressor cells; TAA, Tumor Associated Antigen; TSA, Tumor Specific Antigen; ICD, Immunogenic Cell Death inducers; GM-CSF, Granulocyte-macrophage colony-stimulating factor; IFNs, interferons; ASI, active specific immunotherapy; TKI, tyrosine kinase inhibitor; CTLA-4, Cytotoxic T cell antigen−4; PD-1, Programmed cell death receptor-1; PDL, Programmed cell death ligand.

Radiation therapy in particular, together with its direct cyto-reductive activity on tumor burden is also capable of eliciting radio-induced DNA damage on target cells and triggering specific immunological effects (16) which are believed to be responsible for the “abscopal effects” observed in those rare cases, where tumor irradiation is paralleled by regression of non-irradiated tumor sites (17, 18). This hypothesis is in line with the results of a large number of studies showing that tumor irradiation may really influence all the phases of the immune-response. Tumor irradiation may in fact, trigger immunogenic cell death, and significant release of TAAs and TSAs in a context of immunological danger signal. The latter is consequent to DNA damage by radiation which is able to activate of Damage-Associated-Molecularbiochemical Patterns (DAMP) which in turn are able of enhancing tumor antigens presentation to CTL precursors and their proliferation in the draining lymph-nodes (19). Furthermore, the irradiated-tumor cells release inflammatory cytokines, chemokines (such as CXCL16) and tumor vessel associated adhesion molecules (VCAM-I and ICAM-I) able to reinforce the presence of activated CTLs in the tumor site (19–21). Finally, strong evidence does exists concerning the ability of radiation therapy to induce up-regulation of class I MHC, multiple death receptors (e.g., FAS, NKG2DL) in the target cells thus enhancing their susceptibility to recognition and killing by tumor specific CTLs (19). Clinical evidences in line with these preclinical results have also been reported.

IAn abscopal response to radiation was recorded in metastatic NSCLC patients who were receiving immunological treatment with ipilimumab (22). We recently carried out a retrospective analysis in advanced NSCLC patients enrolled in the BEVA2017, who had received an immune-modulating treatment with metronomic chemotherapy (mPE) +/– bevacizumab (mPEBev) reporting that that the use of radiotherapy given on palliative setting, was associated to a prolonged survival and that this effect was indeed correlated to a significant treatment-related increase in activated DCs and effector memory CTLs (23). Similarly, in a retrospective analysis of the KEYNOTE-001 phase I study aimed to investigate Pembrolizumab in a cohort of 495 patients advanced NSCLC patients, it has been detected a much longer PFS and OS in a group of 97 patients who had received radiation therapy prior immunotherapy (24). Finally, a perspective randomized phase III study in un-resectable lung stage III cancer patients aimed to receive chemoradiation followed by Durvalumab or placebo for 12 months (PACIFIC) reported a significant advantage in PFS in the experimental arm, which was unrelated to PDL-1 expression in the tumor (25).

In HNSCC, the immune system is known to have a pivotal role, as high density of tumor-infiltrating lymphocytes (TILs) is associated with improved outcome of patients (26, 27) while tumor tissues and draining lymph-nodes respectively, present a high density of CTLs expressing PD-1 and regulatory T_reg_s over-expressing CTLA-4; a finding that clearly suggests a high suppressive activity of either peripheral and central immune-checkpoints in these patients (28).

Based on this solid rationale, PD-1 blockade with Nivolumab, Pembrolizumab, and Durvalumab represented a concrete option for the treatment of recurrent or metastatic HNSCC to be investigated. At the present, the results of three large trials in HNSCC patients on or after frontline platinum-based chemotherapy, concur to show an median overall response rate of 11.3–18%, with a median time to progression of 9.7 months and a 32%reduced risk of death at 1 year of (29–33). These encouraging results led to the design of a number of clinical trials which are currently ongoing with the specific aim of combining tumor irradiation with immunological agents and/or immune-check point blockade in patients with advanced HNSCC (see Table 1).

**Table 1:** Ongoing trials testing immunotherapy (IT) in combination with radiation therapy (RT) in patients with NSCLC or HNSCC.

**NCT number**	**Study phase**	**Disease stage**	**Trial design *(Experimental arm)***	**Estimated primary completion date**
NCT03391869	Phase 3	Metastatic NSCLC	Local Consolidation Therapy (RT or surgery) after Nivolumab and Ipilimumab (LONESTAR)	December, 2022
NCT03523702	Phase 2	Locally Advanced NSCLC	Selective Personalized Radio-Immunotherapy for Locally Advanced NSCLC Trial (SPRINT)	August, 2020
NCT03176173	Phase 2	Metastatic NSCLC	Radical-Dose Image Guided Radiation Therapy in Treating Patients with Metastatic NSCLC undergoing IT	June, 2020
NCT03110978	Phase 2	Stage I, selected IIa or isolated recurrent NSCLC	Immunotherapy Plus Stereotactic Ablative Radiotherapy (I-SABR) vs. SABR Alone	June, 2022
NCT03164772	Phase 2	Metastatic NSCLC	Safety and preliminary efficacy of the addition of a vaccine therapy to 1 or 2 checkpoint inhibitors for NSCLC.	March, 2021
NCT03313804	Phase 2	HNSCC and NSCLC undergoing IT	Short-course radiation to a single systemic (non-CNS) site within 14 days of the beginning of IT	June, 2018
NCT02999087	Phase 3	Locally advanced HNSCC front-line	Avelumab and Cetuximab plus RT vs Cetuximab-RT and Cisplatin-RT	October, 2019
NCT03085719	Phase 2	Advanced HNSCC after first line therapy	Immunotherapy (Pembrolizumab) in combination with high dose and low dose radiation therapy	October, 2020
NCT03317327	Phase 1/2	Recurrent HNSCC	Re-irradiation and Nivolumab in loco-regionally recurrent HNSCC	November, 2023
NCT03247309	Phase 1	Recurrent HNSCC and NSCLC	TCR-engineered T Cells in Solid Tumors With Emphasis on NSCLC and HNSCC (ACT engine)	December, 2019
NCT02892201	Phase 2	HNSCC with residual disease	Pembrolizumab after RT in patients with residual disease (biopsy proven)	December, 2018
NCT03247712	Phase 1/2	HNSCC before surgery	Test the safety of neoadjuvant immune-radiotherapy to down-staging HNSCC prior to surgical resection.	December, 2025

On these premises, a rationale use of radiation therapy may be included among the various strategies that could potentially increase the efficacy of immunotherapy at different disease settings. We believe that more successful immune-oncological trials should take in consideration this knowledge to improve their benefit NSCLC and HNSCC patients.

## Author Contributions

All authors listed have made a substantial, direct and intellectual contribution to the work, and approved it for publication.

### Conflict of Interest Statement

The authors declare that the research was conducted in the absence of any commercial or financial relationships that could be construed as a potential conflict of interest.
